# Public engagement with genomics

**DOI:** 10.12688/wellcomeopenres.19473.2

**Published:** 2023-09-18

**Authors:** Anna Middleton, Avery Adams, Hugbaad Aidid, Jerome Atutornu, Daniela Boraschi, Julian Borra, Tuba Bircan, Claudette Burch, Alessia Costa, Anna Dickinson, Ann Enticknap, Catherine Galloway, Francesca Gale, Emma Garlick, Em Haydon, Sasha Henriques, Marion Mitchell, Richard Milne, Jack Monaghan, Katherine I Morley, Milena Muella Santos, Laura Olivares Boldu, Fifi Olumogba, Kate Orviss, Vivienne Parry, Christine Patch, Lauren Robarts, Sam Shingles, Cindy Smidt, Ben Tomlin, Sarah Parkinson

**Affiliations:** 1 Wellcome Connecting Science, Hinxton, England, UK; 2 Kavli Centre for Ethics, Science and the Public, University of Cambridge, Cambridge, England, UK; 3 RAND Europe, Cambridge, England, UK; 4 School of Health and Sport Sciences, University of Suffolk, Ipswich, England, UK; 5 The Thin Air Factory Ltd, London, England, UK; 6 Science and Technology, Onward, London, England, UK; 7 Clinical Genetics Department, Guy's and St Thomas' Hospital, London, England, UK; 8 Melbourne School of Population Health, The University of Melbourne, Melbourne, Victoria, Australia; 9 Public Policy Projects, London, England, UK; 10 Genomics England, Queen Mary University of London, London, England, UK

**Keywords:** Genetics; genomics; public engagement; Responsible Research and Innovation; inclusivity; participation

## Abstract

As detailed in its flagship report, Genome UK, the UK government recognises the vital role that broad public engagement across whole populations plays in the field of genomics. However, there is limited evidence about how to do this at scale. Most public audiences do not feel actively connected to science, are oftenunsure of the relevance to their lives and rarely talk to their family and friends about; we term this dis-connection a ‘disengaged public audience’. We use a narrative review to explore: (i) UK attitudes towards genetics and genomics and what may influence reluctance to engage with these topics; (ii) innovative public engagement approaches that have been used to bring diverse public audiences into conversations about the technology. Whilst we have found some novel engagement methods that have used participatory arts, film, social media and deliberative methods, there is no clear agreement on best practice. We did not find a consistently used, evidence-based strategy for delivering public engagement about genomics across diverse and broad populations, nor a specific method that is known to encourage engagement from groups that have historically felt (in terms of perception) and been (in reality) excluded from genomic research. We argue there is a need for well-defined, tailor-made engagement strategies that clearly articulate the audience, the purpose and the proposed impact of the engagement intervention. This needs to be coupled with robust evaluation frameworks to build the evidence-base for population-level engagement strategies.

## Introduction

Genetics has been a growing area of research and clinical focus, particularly in the United Kingdom over the past decade. In its flagship report, Genome UK, the UK government sets out an ambition for creating “the most advanced genomic healthcare ecosystem in the world”, incorporating both clinical care and research ^1^. This continues the work that started with the creation of Genomics England in 2013 ^2^, which led to the delivery of the 100,000 Genomes Project ^3^ and resulted in the ability to incorporate genomics into clinical care via the introduction of the UK’s National Health Service (NHS) Genomic Medicine Service ^4^, available free at the point of delivery across the entire UK population.

A key feature of the UK government's vision for genomic healthcare is the recognition of the important role of public engagement and dialogue ^1^. Stakeholder and public engagement are “widely lauded as an important methodology for improving clinical, scientific, and public health policy decision-making” ^5^. Public engagement in science (and genetics in particular) is known to contribute towards making sure research is of high quality ^6^, ensuring genetic medicine addresses inequalities in clinical care ^7^, and that the diverse population of the UK is represented, and benefits are shared with segments of the population that have historically been excluded from research ^8^. Failure to engage the public significantly limits the potential societal benefit of genomics. From a public perspective, as per the NHS constitution, everyone has a right to be able to access the benefits of medicine, if they so choose, and be given the opportunity to “influence and scrutinise the planning and delivery of NHS services” ^9^.

However, these rights can only be exercised regarding genomic medicine if there is some level of awareness of *why* we (as publics, patients and future patients) might care about this. This could involve facilitating conversations about what genomics is, what the technology can offer, the relationship between genomic research and clinical practice and discussion around the benefits and challenges so that people can make informed decisions about what they engage with and what services they actually want. We are not advocating that all of society *should* engage with genomics, nor do we actively promote or proselytise the potential benefits. We also respect that even with opportunities to engage, everyone has a right to ignore, opt out or withdraw.

We support the position that science exists to serve society, and this can only happen in ethical ways if publics *(“publics” in plural recognises the heterogeneity of whole populations)* are enabled to make meaning of this for their own lives and opportunities are created to represent public views in policy – whether they choose to take up these opportunities, is up to them. Such policy may be shaped towards the delivery of clinical or research practices and regulatory frameworks.

One in 17 people in the UK will either have or are at risk of having a rare, serious, potentially life-threatening genetic condition ^1^. The definition of “patient” within the context of genomic medicine not only refers to people who have a genetic condition, but also their relatives ^1^ and so even people who are not currently receiving any medical care may be considered a “patient in waiting” ^10^. Thus, genomics is increasingly likely to be relevant to all of us in our lifetime.

People in the UK see the value of science and are interested in it 11, 12, more so than the overall average in the European Union ^12^. However, very few of the UK population feel *actively connected* to science and a third of the British public says they rarely or never talk to their family, friends or colleagues about science ^13, 14^. In this narrative review, we define this lack of active connection as ‘disengagement’, i.e.while people might say they value science, they do not necessarily intentionally seek it out, maintain and build active connections to it or deliberately lean into conversations about it. Thus, to be explicit, when we discuss *broad public engagement*, we are referring to an attempt to connect with a mostly *disengaged-from-science* audience.

The reasons for disengagement are likely to be complex and multi-faceted. However, one confounding effect could be the propensity of science communication and engagement approaches that deliver one-way knowledge dissemination from experts to the public, without specific attention given to nourishing what an *active connection* to science looks like. While science dissemination may work well for public audiences who make intentional choices to seek out traditional teaching about genetics, this style of communication may be insufficient to create positive attitudes towards, and trust in, scientific innovation, particularly for those public audiences who are not already engaged in the subject matter ^15, 16^.

It has long been known that broad deficit models (even though they may be delivered in very creative ways) cannot be the sole solution for helping society become connected to science ^17– 20^. Indeed, established efforts in responsible research and innovation argue for the importance of “dialogic interactions between all actors” in public engagement, allowing publics to contribute their knowledge and experience to research and innovation efforts ^21^. Despite over 30 years of investment in public engagement programmes around genetics and genomics that profess to promote dialogic over deficit models, familiarity, and trust in the science, amongst representative British audiences, remains low ^17^. As genuine dialogue could reasonably offer a forum for debate and airing of concerns, it may be possible to increase trusting relationships between scientists, clinicians and publics through more dialogic approaches to public engagement. This highlights the importance of having clarity of purpose as to why engagement is being done, what it purports to achieve, and a clear evaluation framework that measures whether it has indeed delivered the desired impact. It also pivotal to consider when, in the research pipeline, engagement opportunities should be presented. All too often public engagement is delivered at the end of a translation process once the discovery science has been completed and decisions have already been made about its application. Instead, public engagement can usefully occur before, and within, science pipelines and publics should have an opportunity to co-design and influence the way science is done.

There must be pressure for scholarship in the public engagement space to be published and made easily accessible so that we have the ability to learn and evolve in this field. We are unaware, in the UK specifically, of any consistently used, evidence-based engagement strategies around genomics, nor any detailed strategies that could be used to build long-term coordination of engagement initiatives between different organisations. 

Disengaged public audiences are, by their very nature, the hardest to reach and we define them as those (for whatever reason) who are not actively or intentionally seeking out engagement opportunities, or who when presented with engagement opportunities, look the other way. This is a very heterogeneous group, and possibly a majority of representative publics, including those with a passive interest but limited motivation to engage, those who do not see the relevance of the topic to their lives or who are completely unfamiliar, to those who are cynical and sceptical which causes them to walk away (or at least not step forward). Disengaged audiences are also less likely to participate in genomic studies and might also include those who are described as ‘underserved’, ‘marginalised’ or ‘under-represented’ in both public engagement and also science itself.

An ‘engaged public audience’ makes deliberate choices to walk towards the science, for example by intentionally seeking out research or conversations about genomics because they have a special interest (e.g., they are a genetics patient, an activist or a citizen scientist etc), a specific question, have strong beliefs (including positive or negative) or they are generally motivated and inspired to increase their science capital. Ensuring genomics is of genuine benefit to all of society is highly dependent upon engagement strategies that translate the science in ways that are meaningful for everyone and not just those who are already invested and interested. To extend the metaphor, we must be cognisant of inclusive practices that seek the involvement of the public who are standing still, unsure, unfamiliar, or even walking in the other direction. When thinking about accessibility, we know the content of the engagement needs careful consideration as *what we say* is often different to *what people hear* ^22^ and thus we need to be clear about the impact we aim to deliver if we are to avoid alienating the very people the policy-makers purport to serve.

The need for innovation within public engagement is recognised ^23– 26^ which has led to the showcasing of more participatory and diverse methods of dialogue and public involvement, both in the research itself and in decision- and policy-making. Participation in engagement activities may be constrained by social, political and cultural factors, as well as by (lack of) resources ^21^. We need to ensure that engagement approaches connect with public audiences in ways that move beyond deficit models and meet them *where they are at* ^13, 14^. If we believe that dialogic approaches are important, we need to clearly define how these are delivered so that they have the desired impact, and we need to articulate what this impact is (to change policy? to unpick controversial views? to increase the trustworthiness of science? to increase access to clinical services? to help heal injustice?) It is insufficient to just 'have conversations about genetics’ without being clear about whether and how this is mutually beneficial and evolves the relationship between science and society. And also it is pivotal to understand if innovative public engagement methods are indeed the solution to reaching disengaged audiences. The evidence base to support novel approaches, particularly regarding their effectiveness, is limited ^27^. Thus, more work is needed to investigate this, particularly in relation to effectiveness for groups that have historically felt (in terms of perception) and been (in reality) excluded from genomic research and from scientific studies more widely ^21, 24, 27, 28^.

To ensure equity of involvement in genomic research and access to genomic medicine, and to achieve Genome UK’s vision around public engagement and dialogue, further efforts to engage the UK public with science, health, and genomics more specifically, are needed. We use this narrative review to explore what is known in the peer-reviewed and grey literature about how to do this, where there are gaps in the knowledge base, and formulate suggestions for new research to move this area forward. Our aim was to focus on innovative and novel approaches beyond basic dialogic models and variations of the deficit model, particularly those that seek to engage audiences that are currently not actively engaged, to the extent that they may not be aware of how genomics affects them, how it could be relevant to their lives, or indeed how they feel about ethical issues (e.g., autonomy, privacy, confidentiality). We also sought to better understand the landscape of current attitudes towards genomics, with the assumption that attitudes may play a part in explaining the motivations for disengagement. 

## Methods

The aims of this narrative review were:

- To provide context about public attitudes towards genetics and genomics and their technological applications;
- To describe and provide a sense of the scale of audiences that are disengaged from genetics and to provide an explanation for this disengagement to the extent possible;
- To understand which innovative forms of public engagement have been used to connect public audiences to the science of genetics and why and how its application is relevant in their lives.

Thus, our overall objective was not to determine whether a particular approach is effective, but to develop an understanding of the context(s) in which novel approaches have been developed, and how they have been used in practice. Consequently, we conducted an interpretive narrative review, drawing on the approaches used in *hermeneutic reviews* and *critical interpretive synthesis* ^29– 31^. We chose this approach to give us the broadest overview possible of a niche field that uses different methodological approaches in both research and engagement delivery. 

We did not seek to provide a comprehensive and systematic review of studies in these areas, but rather to describe, in broad terms, the types of peer-reviewed research and engagement that have been undertaken to make recommendations for further areas of development. However, we used a structured approach to identifying relevant literature based on the following criteria:

- A focus on either “genetics” or “genomics”. Although we use the term genetics to refer to single gene testing, and genomics to refer to whole genome approaches to research or clinical testing.
- A focus on *human* genetics, as opposed to other species or agriculture.
- A focus on “public” that included whole “populations” as well as those who are “underserved”, “marginalised”, “underrepresented” or “disengaged”.
- A focus on “public engagement” approaches described as “novel”, particularly where these seek to engage with people who may not usually be included in engagement efforts.
- Published in 2017 or later, given the rapid progress made in the field of genomics in the past five years, and the degree to which the general public has become more aware of the field over time.

While our focus is on the UK, international evidence is included where it does not focus on a specific country. Given the public engagement landscape is very broad internationally, with no universally accepted definition of public engagement itself, we wanted this specific narrative review to start with the literature most meaningful to the UK context. However, given that novel engagement methods are infrequently labelled as ‘novel’ in the literature, we included those that were indexed with this term, regardless of country. See Table 1 for inclusion and exclusion criteria.

**Table 1.** Inclusion and exclusion criteria.

	Inclusion	Exclusion	Search		
			*Attitudes*	*Disengaged* * populations*	*Public * *engagement*
**Date**	2017 and later	2016 and earlier	✓	✓	✓
**Language**	English	Non-English languages	✓	✓	✓
**Population**	General population with no engagement in genetics/genomics	Specific populations already involved in genetics/genomics (e.g., early adopters of DTC genetic testing, people involved in research or genetic studies, people already receiving genetic tests in clinical care, patients)	✓	✓	✓
**Country**	All countries	N/A			✓
**Country**	UK International studies including the UK or not specifically focused on another country	Focus on particular non-UK country	✓	✓	
**Study design**	Any other type of publication (empirical studies, primary studies, reviews, perspectives or opinion pieces, case studies)	Abstracts Protocols Marketing material for DTC companies Clinical practice guidelines Case reports Clinical trials Conference proceedings	✓	✓	✓
**Intervention**	Novel types of engagement (e.g., film, video, music, theatre, dialogue, social media engagement)	Traditional types of engagement (e.g., teaching, brochures, lectures, posters – anything that just gives people information without additional activity)			✓
**Outcomes**	Perspectives about genetics/ genomics (e.g., attitudes, beliefs, willingness to take test)	Studies looking at behaviour (whether or not test is taken) Perspectives about (positive or negative) results of genetic testing General like/dislike of genetics or science	✓		
**Outcomes**	Who is engaged in genetics/ genomics Whether or not historically disengaged populations participate in genetics/genomics Willingness to participate in genetics/genomics Change in willingness or actual participation in genetics/genomics	Other outcomes not related to perspectives about genetics/genomics, willingness to engage or actual engagement		✓	✓

### Search strategy

Three separate searches were conducted in Scopus and PubMed, each with a different focus (see Table 2):

**Table 2.** Search terms and number of results for academic literature.

SEARCH	SEARCH TERMS AND RESULTS
**Search 1 – Public attitudes** ** towards genetics/genomics**	**PubMed: Returned 131 hits** ((“genetic*”[Title/Abstract] OR “genomic*”[Title/Abstract] OR “precision medicine”[Title/Abstract]) AND (“public opinion*”[Title/Abstract] OR “public attitude*”[Title/Abstract] OR “public interest*”[Title/ Abstract] OR “public view*”[Title/Abstract] OR “public acceptability”[Title/Abstract] OR “public perception*”[Title/Abstract] OR “social attitude*”[Title/Abstract] OR “social acceptability”[Title/Abstract]) AND ((y_5[Filter]) AND (humans[Filter]) AND (english[Filter])) ) NOT (agricult*) **Scopus: Returned 125 hits** (TITLE-ABS(genetic*) OR TITLE-ABS(genomic*) OR TITLE-ABS(“precision medicine”)) AND (TITLE- ABS(“public opinion”*) OR TITLE-ABS(“public attitude”*) OR TITLE-ABS(“public view”*) OR TITLE- ABS(“public interest”*) OR TITLE-ABS(“public acceptability”*) OR TITLE-ABS(“public perception”*) OR TITLE-ABS(“social attitude”*) OR TITLE-ABS(“social acceptability”*)) AND NOT (TITLE-ABS-KEY(animal*) OR TITLE-ABS-KEY(agri*) OR TITLE-ABS-KEY(food)) AND ( LIMIT-TO ( DOCTYPE,”ar” ) OR LIMIT-TO ( DOCTYPE,”re” ) ) AND ( LIMIT-TO ( SUBJAREA,”SOCI” ) OR LIMIT-TO ( SUBJAREA,”ARTS” ) OR LIMIT-TO ( SUBJAREA,”PSYC” ) OR LIMIT-TO ( SUBJAREA,”MEDI” ) ) AND ( LIMIT-TO ( PUBYEAR,2022) OR LIMIT-TO ( PUBYEAR,2021) OR LIMIT-TO ( PUBYEAR,2020) OR LIMIT-TO ( PUBYEAR,2019) OR LIMIT-TO ( PUBYEAR,2018) ) AND ( LIMIT-TO ( LANGUAGE,”English” ) )
**Search 2 -Disengaged** ** populations**	**Pubmed: 44 hits** (“genetic*”[Title/Abstract] OR “genomic*”[Title/Abstract] OR “precision medicine”[Title/Abstract]) AND (“public”[Title/Abstract] AND (“disengage*”[Title/Abstract] OR “underserv*”[Title/Abstract] OR “margin*”[Title/Abstract])) AND ((y_5[Filter]) AND (humans[Filter]) AND (english[Filter])) **Scopus: 65 hits** ( TITLE ( genetic* ) OR TITLE ( genomic* ) OR TITLE ( *“precision medicine”* ) ) AND ( TITLE-ABS-KEY ( *public* ) AND ( TITLE-ABS-KEY ( *disengag* ) OR TITLE-ABS-KEY ( *underserv* ) OR TITLE-ABS-KEY ( *margin* ) ) ) AND ( LIMIT-TO ( DOCTYPE , *“ar”* ) OR LIMIT-TO ( DOCTYPE , *“re”* ) ) AND ( LIMIT-TO ( PUBYEAR , *2022* ) OR LIMIT-TO ( PUBYEAR , *2021* ) OR LIMIT-TO ( PUBYEAR , *2020* ) OR LIMIT-TO ( PUBYEAR , *2019* ) OR LIMIT-TO ( PUBYEAR , *2018* ) OR LIMIT-TO ( PUBYEAR , *2017* ) ) AND ( LIMIT-TO ( LANGUAGE , *“English”* ) )
**Search 3 – Novel public** ** engagement methods**	**Pubmed: 31 hits** ((“genetic*”[Title/Abstract] OR “genomic*”[Title/Abstract] OR “precision medicine”[Title/Abstract]) AND (“public engagement”[Title/Abstract] OR “public outreach”[Title/Abstract]) AND ((y_5[Filter]) AND (humans[Filter]) AND (english[Filter])) AND ((y_5[Filter]) AND (humans[Filter]) AND (english[Filter])) ) NOT (agricult*) **Scopus: 370 hits** ( TITLE-ABS ( genetic* ) OR TITLE-ABS ( genomic* ) OR TITLE-ABS ( “precision medicine” ) ) AND ( TITLE- ABS-KEY ( *public* ) AND ( TITLE-ABS-KEY ( *engagement* ) OR TITLE-ABS-KEY ( *outreach* ) ) ) AND ( LIMIT-TO ( DOCTYPE , “ar” ) OR LIMIT-TO ( DOCTYPE , “re” ) ) AND ( LIMIT-TO ( PUBYEAR , 2022 ) OR LIMIT-TO ( PUBYEAR , 2021 ) OR LIMIT-TO ( PUBYEAR , 2020 ) OR LIMIT-TO ( PUBYEAR , 2019 ) OR LIMIT- TO ( PUBYEAR , 2018 ) OR LIMIT-TO ( PUBYEAR , 2017 ) ) AND ( LIMIT-TO ( LANGUAGE , “English” ) )

- Search 1 reviewed public attitudes towards genetics and genomics.
- Search 2 reviewed the characteristics of populations that are disengaged from genetics and genomics and potential reasons for this disengagement.
- Search 3 reviewed innovative public engagement methods that have been used to connect disengaged audiences to genetics and genomics.

All academic literature searches were conducted on 16 July 2022, and were limited to humans, English-language literature, articles published 2017 or later, and reviews and standard articles. Grey literature searches were also conducted in Google Scholar and Google ( Table 3). The first 100 most relevant results were reviewed in Google Scholar, and the first 50 most relevant results were reviewed in Google. As part of the grey literature search, we also searched the Participedia website for examples of public engagement in genomics. Participedia is an online platform that collects case examples of public participation and democratic innovations internationally, along with examples of methods and procedures used in public engagement and organisations that focus on participatory governance. We included results from this search that fit the inclusion criteria for the overall review.

**Table 3.** Search terms for grey literature.

SEARCH	SEARCH TERMS AND RESULTS
Search 1 – Public attitudes towards genetics/genomics	(genetic OR genomic OR “precision medicine”) AND (“public attitudes” OR “public opinion” OR “public opinions” OR “public perception” OR “public perceptions” OR “social attitude” OR “social attitudes”) AND (England OR UK)
Search 2 – Disengaged populations	(genetic OR genomic OR “precision medicine”) AND (“disengaged” OR “underrepresented” OR “underserved” OR “marginalized” OR “marginalised”) AND (“groups” OR “populations” OR “public”) AND (England OR UK)
Search 3 – Novel public engagement methods	(genetic OR genomic OR “precision medicine”) AND (“public engagement” OR “public outreach”) AND (“innovative” OR “novel” OR “creative” OR “arts-based” OR “non-traditional” OR “interactive” OR “participatory” OR “online”

### Screening

The search process produced 624 articles after de-duplication. These articles were single-screened by three team members in Rayyan ^32^ using the exclusion and inclusion criteria in Table 1. A total of 23 articles from the academic searches met the inclusion criteria, in addition to 21 grey literature articles. Thus 44 articles were initially taken forward for data extraction. In the extraction phase we identified 33 additional sources through “snowballing”, which were incorporated into the review. Searching the Participedia website also resulted in two further examples of public engagement in genomics, which were included in the analysis. In total, 79 sources were included in this narrative review.

In line with a hermeneutic circles approach ^30^, we initially included all studies that were described by the authors as “public engagement” but in reviewing the included studies, we distinguished between those that aimed to facilitate bi-directional communication between publics and professionals or experts and those that sought only to facilitate uni-directional delivery of information from experts to publics ^20^. We attempted to focus on papers reporting novel approaches to public engagement, particularly where these were focused on engagement with groups not normally involved in research and innovation. In conducting the searches, we relied on the authors’ definition of “novel” to identify articles but considered this in a more nuanced way when screening and reviewing papers.

### Extraction, analysis and synthesis

Data was extracted from the included articles that were included in this narrative review using a coding framework in MaxQDA ( Table 4) ^33^. This coding framework was developed based on the aims of this narrative review and used to map and classify the findings from the literature ^30^. Once coding in MaxQDA was completed, the coding framework was used to structure analysis and synthesis, supporting a critical assessment of the literature ^30^. The results of this analysis and synthesis are reported below.

**Table 4.** Coding framework for narrative review.

CODE		
Information about study	Publication type	Other
		Primary study
		Review
		Perspective
	Sample characteristics	
	Method	
	Other	
Population	General population	
	Ethnicity or nationality	
	Gender	
	Age	
	Sexual orientation	
	Socioeconomic and education	
	Knowledge of genetics	
	Other	
Types of genetics activity	Genetics in research	
	Genetics in clinical care	
	Genetics (general)	
	Other	
Attitudes	Awareness	
	Understanding or knowledge	
	Trust	
	Willingness to participate	
	General attitudes	Positive
		Negative
		Other	
	Other	
Disengaged populations	Reasons for disengagement	
	Groups that are disengaged	
	Other	
Public engagement	Art	
	Film	
	Music	
	Theatre	
	Social media	
	Other	

## Findings

### Public attitudes towards genetics and genomics and their technological applications

International evidence, including large-scale systematic reviews, shows that knowledge, understanding and awareness of genetics is limited among the general population in many countries, including the UK ^34– 38^. Research suggests that the link between genetics and health is not well understood, including concepts such as genetic susceptibility to conditions like cancer ^11, 12^, the distinction between conditions caused by multiple genetic and environmental factors as opposed to a single genetic variant, and the role of genetics in contributing to traits such as weight and educational attainment ^38^. Knowledge of genetics varies in the general population; women, people who are older, have a lower income, have less formal education and live in more rural areas tend to have less knowledge and awareness of genetics ^34, 38, 39^. Groups who identify as being from a minority ethnic group in the UK also tend to have less knowledge of genetics ^17, 34, 35^. However, although knowledge and understanding of genetics and genomics is generally low amongst the general population, many public audiences have nuanced perspectives of clinical care and research and see benefits of the technology in terms of prevention and treatment of disease ^34, 35, 40, 41^.

### Attitudes towards the use of genetics and genomics in clinical care

 Positive attitudes regarding the clinical application of genomics are generally linked to a belief that they contribute to better health outcomes, and improvements in medicine and treatments ^34, 35, 41– 43^. Similarly, positive attitudes to research can stem from a belief that participation in genomics will help family members ^35^ and society more widely ^35, 44^, help individuals prepare for the future ^45^ or inform decision-making (e.g., around reproductive health) ^43^. In general, those that see contributing to genetic studies as important in creating scientific and medical progress have more favourable views about the science ^20, 40, 46^.

Although enthusiasm around genetic testing within the context of healthcare and genomic medicine tends to be high overall ^8, 39, 41, 43, 45, 47^, perceptions of genetic testing for inherited disease may vary, based on the condition being tested for and the test itself. For example, public audiences view genetic testing for heritable conditions, genetic variants with high penetrance, and tests for more debilitating diseases (e.g., Huntington’s Disease) more favourably ^8, 34, 38– 40, 47^ as well as conditions that are treatable or preventable ^34, 39, 40, 43, 47^. Tests perceived as having a higher predictive ability and those that indicate large increases in disease risk are similarly preferred over tests that predict small increases in lifetime risk of illness ^39^. On the other hand, genetic tests perceived as being for non-medical purposes (e.g., testing for cosmetic traits or intellectual advantage) are viewed less favourably ^8, 40, 47^. Some research has shown public concern that predicting future disease may cause psychological and emotional harm, contributing to an unwillingness to engage, particularly amongst members of ethnic minority groups in the US and UK ^34, 35^. These concerns may be amplified where tests are conducted antenatally or on infants ^43^.

With respect to applications that are only emerging as part of clinical care, such as gene editing and gene therapy, some research has highlighted concerns around adverse side effects, risks, and unpredictable long-term consequences, along with existential fears over human genetic diversity and the implications of increased life spans which some associate with gene editing ^8, 42, 44^. Perhaps for this reason, somatic therapies are seen as more acceptable than germline therapies ^8, 47^. Costs are also a cause for concern ^34, 35^, particularly if high costs lead to inequalities in access to genetic modification therapies ^48^. This is a concern particularly in the US context due to a lack of universal health coverage ^35^.

### Attitudes towards genetics and genomics research

Attitudes toward genetic and genomic research, as opposed to its use in providing clinical care, are more complex. Overall, those who are less familiar with genomics seem to be less willing to participate in studies that collect genetic data and have lower levels of trust in genetics research ^17, 38, 42, 47, 49^. However, high levels of understanding or knowledge do not necessarily lead to positive attitudes toward genetics ^24^. High knowledge levels can lead to particular concerns and worries not present in those with less knowledge – for example, concerns about how data are used ^23, 50^.

People in England are generally willing to have genetic data shared in a healthcare context and for this to be used for research activities ^44^, but not to the same extent as for the application of genetic and genomic technologies to deliver clinical care ^17^. Negative attitudes towards genomics generally stem from concerns around data use, confidentiality, and privacy ^17, 34– 36, 44, 48, 49, 51^, and incidents in which the NHS failed to securely store data may play into fears around mishandling of data ^44^. Indeed, members of the public with more personal experience of genetics in relation to health and disease in their family ^17, 35, 38– 40, 49^ have more positive attitudes toward genetics and genomics but also have more concerns about how genetic data are used. There are also fears over genetic discrimination (e.g., from employers or insurers) ^17, 34, 35, 41, 47– 49^, and concerns around the perception that DNA data can be used against individuals, for example, if it were to be planted at the scene of a crime ^17, 46^.

Given these concerns, it is not unexpected that willingness to donate genetic samples and positive attitudes around genetics are associated with trust in those that use genetic data ^36, 40, 47– 49^. Medical doctors are generally viewed as the most trustworthy custodians of these data while for-profit companies are generally viewed as the least trustworthy ^17, 40, 49^. Milne *et al.* (2019) found that across 37,000 members of the public from 22 countries, there were significant numbers who do not trust anyone with genetic information other than their medical doctors ^49^. This creates challenges in terms of who can facilitate the storage and sharing of genetic data ^47, 49^. People may be more willing to approve the use and reuse of genetic data where explicit consent is obtained ^44, 51^, safeguards are in place ^40^, and where these data are made available in a safe haven with strong governance rather than an open access environment ^51^. 

### Audiences disengaged from genetics and genomics and possible reasons for this

Within the general population, there are people from specific socio-demographic groups who are less willing to participate in genomic studies and have negative attitudes and concerns towards the science. These include women, people who are older, have lower income, have no tertiary education, and self-identify as belonging to a minority ethnic group ^8, 17, 35, 49– 55^. The sources of negative attitudes to genetics and genomics appear to be related to mistrust of the technology based on cultural or shared beliefs or historical examples of unethical research, the potential for genetic discrimination, and lack of appropriate representation for marginalised groups ^6, 17, 35, 49– 55^.

### Cultural or shared beliefs

 Cultural contexts and shared beliefs can influence attitudes towards genetics and genomics. Cultures with spiritual or supernatural beliefs may use these to explain disease ^46^ which can conflict with genetic explanations. In some cultures, genetics may also be viewed as a “Western medicine” ^35, 52^ and there may be taboos or stigmas around discussing diseases with a genetic basis such as cancer ^35^. Some Asian-American and Chinese-Australian participants in studies about genetics expressed concern that receiving a genetic test would create a stigma of “bad genes” which could impact marriage prospects ^35^. There is also some limited evidence that people who self-identify as having religious beliefs are less likely to find gene therapy acceptable ^17, 47^.

Both misinformative “anti-science” representations ^40, 50, 56^ and real accounts of scientific exploitation ^6, 37^ can contribute to mistrust in genetics and genomics. Media representations of genetics (both real and fictional) also influence how public audiences feel about genetics ^6, 35, 40, 44^, as do advertisements for Direct-to-Consumer testing ^45^. There is substantial evidence that the history of racism and discrimination in science has had a significant impact on the willingness of ethnic minority groups to participate in research ^57^. For example, participants in a US study about giving consent for the use of genetic data cited the Tuskegee Syphilis study (an unethical study which began in 1932 where Black men were knowingly infected with Syphilis), with one South Asian participant citing “the way minorities have been used in research in this country and [how] that history kind of still definitely permeates” ^58^. In another study, Henrietta Lacks—an African American woman whose cancer cells were used as the source of the HeLa cell line without her knowledge, and who was never compensated—was mentioned by publics across different ethnic groups ^59– 61^.

### Potential for discrimination

 People from different population groups have expressed concerns about the application of genetics and genomics due to misrepresentation of research findings or discrimination. In the US, African American and Latino populations had significantly higher rates of mistrust in genetic testing than self-identified white American populations and were more likely to believe that results could be used to depict their ethnic group as inferior ^35^. Also in the US, African Americans have expressed concerns that genetic tests will not be accurate for them, while indigenous peoples have expressed reluctance to participate in genomic research because of their fears around the potential for the commodification of drugs based on their genomic data ^35, 53^. Similar findings have been reported in the UK, where self-identified Black Caribbean and Black African members of the public have expressed suspicion and mistrust of the 100,000 Genomes project ^1^ and members of the Pakistani community have been identified as having a fear of genomic information and mistrust of genomic research ^62^.

There is some evidence available on what drives the exclusion of certain groups from genomics research in the UK. Mulrine *et al*. (2021) conducted ethnographic fieldwork in Northeast England and found that asylum seekers, those experiencing domestic violence, transgender people, offenders, and ex-offenders had serious concerns about health-related data sharing ^63^. For these individuals, concern about genomics was not rooted in a lack of awareness or understanding, but in negative experiences of healthcare discrimination and in an awareness of the risks of data sharing specific to people from marginalised backgrounds ^63^. LGBT+ participants in particular did not perceive NHS services as being fit to handle sensitive and potentially stigmatising information ^63^.

### Lack of representation

 Internationally, many groups have been identified as being historically under-represented in research relating to genomics (i.e., by virtue of their lack of representation they are structurally and practically disconnected or “disengaged” from genomic research, whether by choice or not). These include people from low- and middle-income countries, indigenous peoples, and minority groups including sexual, gender and ethnic minorities ^6, 52, 54^. There is also some evidence for greater mistrust of genetics among the D/deaf and hard-of-hearing ^64^ and sexual and gender minorities, with the latter two groups expressing concerns that questions asked in the research would not accurately capture their behaviours or identity ^52^.

The disproportionate focus of research on certain groups is pervasive. Recent analysis of the Genome Wide Association Studies (GWAS) Catalogue estimated that 86% of genomics studies have been conducted on individuals of European descent and that the proportion of studies conducted with under-represented populations has been stagnating or decreasing ^6^. There are also inequalities in terms of geographic representation, with 90% of enrolled participants in the recent International HundredK+ Cohorts Consortium study being from North America or Europe ^6^. UK Biobank predominantly contains data from participants who self-identify as White British (94.6% of the dataset). While this is similar to the UK population (estimated to be 91.3% White in 2011) ^65^, there is still a need for other dimensions of diversity, for example, in relation to socio-economically deprived publics and those with self-reported health conditions ^6^. Public audiences are aware of this lack of representation and the long-term impact it may have on clinical care; in a recent public dialogue on whole genome sequencing for newborn screening in the UK, participants from Black, Asian and Minority Ethnic (BAME) groups expressed concern that under-representation in genetic research would contribute to continued inequality in healthcare provision ^66^. They agreed that a comprehensive genetic database should be established so that people from minority ethnicities can benefit from newborn screening (e.g., by ensuring that tests are accurate for them) ^66^.

### Scale of disengagement

As described in our Introduction, we have defined “disengaged public audiences” as those who are not actively seeking out engagement opportunities, or who when presented with engagement opportunities, look the other way. Of course there are ‘engaged public audiences’ who feel actively connected to the science, but their attitudes towards it are profoundly negative. Thus having negative attitudes towards genetics could be a driver for seeking out engagement opportunities with the purpose of complaining, demonstrating or challenging the status quo. Actively seeking out engagement opportunities does not equate to only having positive attitudes towards the science.

We have described some of the attitudinal reasons why publics may feel concerned or positive about genomics and infer that this may be associated with engagement seeking behaviour and more research is needed to more fully understand the direction of this relationship.

We have shown that there are people from specific socio-demographic groups who have less knowledge about genomics and more negative attitudes toward its application (women, people who are older, have lower income, have no tertiary education, and self-identify as belonging to a minority ethnic group ^8, 17, 34, 35, 38, 49– 55^). We have also shown that only a minority of the British public “feel connected to science” and just over a quarter feel that “science is not for me” ^14^. What we don’t yet know is the exact scale of the disengaged audience and can only postulate that it may be the majority of representative publics. More research is needed adequately stratify this very heterogeneous group. 

### Use of innovative forms of public engagement in genetics and genomics

We have discussed some of the attitudinal reasons members of the public may not engage with genetics in research or healthcare contexts. Efforts to deliver public engagement are ongoing, but the research we have summarised above suggests that new approaches may be needed to connect with groups who have limited awareness of, or negative perspectives towards genomics, or who are ambivalent or apathetic about it.

We therefore searched for novel approaches to public engagement in genetics and genomics (bearing in mind that there are hundreds of references to “public engagement” but we specifically wanted to seek out innovation in this space). In conducting this search, we were reliant upon authors’ determinations of whether the reported efforts constituted public engagement, and also whether they could be considered novel or innovative. We were also reliant on engagement practitioners and scholars publishing their work in a searchable format. We found that not all efforts labelled “public engagement” moved beyond the deficit model to do more than simply provide information about genomics. While we report our findings across all the studies we identified, we draw out the degree to which the novel methods identified in the literature promote bi-directional communication between publics and other stakeholders in genetics and genomics. This focus on two-way communication aligns with the definition of public engagement from the National Co-ordinating Centre for Public Engagement:

*“Public engagement describes the myriad of ways in which the activity and benefits of higher education and research can be shared with the public. Engagement is by definition a two-way process, involving interaction and listening, with the goal of generating mutual benefit.” ^67^*

Despite searching both the published peer-reviewed literature as well as the grey, non-peer-reviewed literature, we only identified 17 examples of innovative methods being used for public engagement in genomics since 2017. We have grouped our findings by the type of engagement approaches used: social media and virtual platforms, arts-based (including film and theatre), writing, and scenario-based. However, we acknowledge that these boundaries are artificial as these categories need not be mutually exclusive.

There were also examples of deliberative events that fall outside of these groups. For example, there is ongoing work by the Centre for Deliberative Democracy and Global Governance at the University of Canberra to organise a Global Citizens’ Assembly on Genome Editing ^68^. This will be based on national citizens' juries; to date these have already been delivered in Australia and the UK – both citizens' juries were delivered over four days and involved intense, facilitated debate between members of the public and experts in the ethical, legal and social implications of gene editing. The aim was to create shared meaning on the societal impact of the science that would lead to the creation of policy recommendations for government ^69^.

***Social media and virtual platforms*.** As social media increases in popularity, more studies are using online platforms and virtual means to engage the online public in conversations about genomics ^48, 70, 71^. Some studies we identified used social media and other virtual platforms simply to invite debate and raise awareness of genomics ^48, 70, 71^, while others collected data through social media or asked participants to interact with videos or films through a virtual platform ^70, 72^. Often, this form of engagement has been used to complement more traditional social sciences research (e.g., surveys, interviews), or traditional forms of engagement (e.g., a panel discussion involving a group of experts, and public audiences invited to attend and ask questions) ^70, 72^.

Compared to other “novel” methods of engagement (see below), social media or online-based engagement methods have the widest reach in terms of audiences engaged ^48, 70, 71^. Furthermore, many social media platforms have inbuilt methods of tracking users, allowing the interactions to be measured ^48^. Studies using social media or online platforms to engage their audiences typically can reach people in the thousands or tens of thousands ^48, 71^, but may not always generate meaningful, bi-directional communication with all the audiences they reach.

For example, one study used a Facebook advertising campaign to inform members of the public in Michigan about children’s dried blood spots being stored in a biobank. Those who interacted with advertisements were directed to a Facebook page, where they were encouraged to provide their views about the biobank. Moderators also participated on the page by answering factual questions, correcting or clarifying information, and providing information about opting out of biobanks. Moderators had the goal of maintaining a neutral tone and consisted of the lead researcher and other members of the study team. The campaign was able to reach 1.88 million Facebook users and actively reach around 38,000 people ^48^. However, although this study encouraged conversation between participants, it is unclear the degree to which meaningful engagement, particularly between researchers and members of the general public was accomplished. This study represents public engagement efforts conducted by a third party, in this case, researchers who were not involved in the biobank. The authors noted that this approach is valuable in that members of the public may have been more likely to trust the study, as the invitation to participate did not come from the biobank itself. The researchers did not state what they intended to do with the findings, but the collated public attitudes around consent, privacy, trust, and motivations for participation could be useful in informing future biobank activity. 

In another study conducted by Sciensano (the national public health institute of Belgium), an online discussion platform was created for members of the public in Belgium to discuss ethical, legal and social issues associated with genomics ^71^. Before entering the deliberative platform, participants were provided with an information booklet and a short video about genomics and participated in an interactive scenario illustrating what the world might look like if everyone had the same opinions about genomics that they did. The platform allowed citizens to interact and debate anonymously – they could post their opinions, as well as comment on and discuss opinions shared by other participants. A researcher moderated the platform (e.g., for offensive language and trolling), although it is unclear if they participated in conversations beyond moderation. Overall, the platform gathered 1247 opinions, and allowed for clear two-way communication between participants. The public engagement activity in this study was conducted by the researchers themselves, with the goal of gathering public opinions on genomics in Belgium. Whilst this goal is clear, it is not explicitly stated what the researchers intended to do with the findings and the degree to which the findings actually influenced decision- or policy-making. The authors of the study, however, articulated that the findings could inform future research efforts, particularly in terms of incorporating responsible research and innovation approaches or a “constructive technology assessment” which draws on the perspective of public and social actors to inform technological development into genomics ^72^.

***Arts-based engagement*.** Art has more frequently been used as a public engagement method in genetics and genomics over the past two decades ^20, 24, 73^, although art-based engagement strategies are still largely under-explored and under-researched. From the literature, it is evident that what qualifies as an art-based engagement strategy is defined broadly ^20, 73, 74^ encompassing, for example, interactions with museum exhibitions related to genomics ^73^, performance, conceptual and three-dimensional art ^20, 24^, and participants creating art themselves. Arts-based engagement methods have been used within genomics and also in wider science communication strategies ^20, 73^.

Art encourages engagement in genetics and genomic studies by allowing for personal reflection and interaction and increasing the visibility of genomics ^20, 73^. Museum exhibits (in arts or science museums), for example, have been used to promote genomics to an audience that may face barriers connecting with it due to the complexity of the subject matter ^20, 24^ by simplifying the topic and encouraging a sense of self-efficacy. These types of exhibits can also encourage engagement by incorporating interactive elements (e.g., through gamification) ^73^. However, none of the studies we identified on arts-based engagements clearly demonstrated a two-way dialogue between the public experiencing the art and the researchers or artists communicating about genomics. We can assume that the art elicited some form of dialogue amongst its audience, but the extent to which this happened is not explicitly captured.

For example, a study carried out by Howell *et al*. ^73^ used semi-structured interviews with educators who worked in museums that had genetic exhibitions on display to try and understand the learning goals of the exhibits. Whilst the interviews provided useful insights about the driving force behind the creation of the exhibitions (e.g., being able to communicate that genomics is not a concept that is removed from everyday life), there was no clear process to support dialogue amongst museum visitors. This is a gap that could be addressed in future studies. The interviews for this study were carried out by academic researchers, although the director of science communications at the museum was also involved in the journal article that resulted from the study (alongside the researchers). Individuals in other education and engagement-facing roles in the museum were participants in the study. The authors state that the findings of the study can be used to inform exhibit design that effectively engages the public and increases genetic literacy, although they do not state the extent to which findings were taken up by the museum ^66^.

Although there is increasing interest in the use of film and theatre to engage the public with genetics and genomics ^21, 43, 75^ it is still not very commonly seen in the published literature. Studies that have used film and theatre typically involve a small group of participants and collect qualitative data about their thoughts and opinions on a particular film or play about genomics ^46, 75^, rather than encouraging engagement between experts and the public. This form of engagement has been used to generally target non-expert audiences, which may require adaptation depending on local contexts and language preferences.

For example, a study in South Africa delivered a play about genomics, adapting a script written for an American audience. The language of the play and its contents were adapted to resonate with South African people more closely by changing character names, adding local colloquialisms, culturally adapting scientific jargon, adapting cultural references, and not emphasising individual privacy as heavily ^46^. In attempts to meaningfully engage participants on the subject matter, the play relied on audience members to act in the play, which was followed by a discussion about the various ethical dilemmas surrounding genomics posed within the play. This activity led to a successful dialogue between the study team and participants, and more reflection among participants on genomics. For example, participants reported that the play elicited new questions about the wider genomic space and allowed them to reflect on the perspective of different characters in the play ^46^.

The study and play were conducted by academic researchers, and discussions were facilitated with audience members as well as the researchers themselves, which demonstrated clear two-way communication. While the article does not provide information on how findings from the study will be used, the authors state that the context for conducting the study was in relation to challenges they faced in their collaborative centre (H3Africa ELSI Collaborative Centre), focused on the evidence base for returning individual genetic findings in African genomics research. This implies that the findings may be used to inform how the centre conducts activities with the public, and the authors note that there are also implications in terms of using more plays adapted to African audiences to engage lay audiences in genomics.

Overall, studies involving arts-based engagement methods have found that they are somewhat effective in engaging lay audiences ^20, 73^ and have the potential to reach thousands of people ^20, 24, 73, 74^. However, there is not much clarity about how the information being communicated is interpreted by different audiences or about the impact of these engagement methods ^20, 74^. Fraaije, A. *et al*. note that future research should reflect on outcomes of arts-based engagement, including the extent to which they can change attitudes and behaviours toward genomics ^20^.

***Writing*.** The strength of writing-based engagement strategies is that they offer an opportunity to gather deeply personal reflections ^76^ as well as insights into barriers that people may face in engaging with genomics ^42^, however, they involve one-sided reflection as opposed to two-way dialogue.

For example, a 2006 project (a Mass Observation directive called “Genes, Genetics and Cloning”) ^77^, gathered information from more than 200 participants by asking them to write about their views and understanding of genomic science and the impact that media such as news, films and books has had on their views. This information was then archived and accessed by researchers at later points for analysis ^40, 76^. The researchers commissioned but did not directly run the Mass Observation directive themselves. The researchers did not report how the findings were used and described the data-gathering exercise in terms relating to observation of public opinion, rather than having a specific use. However, they noted that the same Mass Observation directive should be conducted again in the future to assess changes in understanding, engagement, and attitudes towards genomics among the public. Similarly, a 2009 project titled “The Human Genre” used a website to collect short poems inspired by the Human Genome Project. This project has captured the interest of researchers globally as a potential case study of how poetry can be used as a method to humanise genomics and engage with populations that have been excluded from genetics and genomics ^52^. This use of writing as a form of public engagement was similarly focused on observation and understanding of public opinion, rather than two-way dialogue; it is also unclear if it had any impact on policy.

Participatory approaches to writing have also been used in public engagement around genomics. A UK-based study on experiences with rare genetic conditions consisted of a participatory writing programme for families affected by genetic conditions in which the participants wrote about their experiences and participated in workshops and reflective interviews with the aim of building confidence in expressing lived experiences through writing. The study revealed useful insights about the wider impacts of genomics beyond the clinical aspects. The researchers did not state explicitly what they intended to do with the findings from this study, but they reflect that their findings “might prompt greater understanding of the lived experiences of families whose lives have become entwined with the genomics agenda.” ^76^

Although gathering these types of projects certainly support reflection and engagement, it is not well documented to what extent writing-based engagement strategies have prompted dialogue amongst the target audience or between participants and researchers. The Mass Observation directive (described above) had the potential to support dialogue between researchers and respondents ^40, 76^, however, it is not clear whether there was an avenue for respondents to receive any communication in response to their writing. Furthermore, the collection of poems in “The Human Genre” project ^77^ demonstrates how each individual poem can be interpreted by researchers and can prompt conversations about public perceptions of genomics. And there is no mention of how these poems might create dialogue between participants, or between participants and researchers. 

***Scenario-based engagement*.** Engaging the wider public using scenario-based discussions is a strategy that has been widely used within research in general and is now becoming increasingly popular within genomics ^56, 78^. For example, a study by Lehoux *et al.* ^78^ provided participants with fictional scenarios about interventions for genetically at-risk individuals, which were designed to help participants empathise and think through moral- and governance-related issues. Data were collected through workshop discussions and an online platform where participants could comment on one another’s reactions to the scenarios. The public engagement activity for this study was conducted by a professional moderator, and the overall study was conducted and analysed by academic researchers. The researchers noted that the likelihood of their findings being taken up by policy-makers was unclear, although it resulted in relevant findings that could potentially inform decision making relating to the ethical dilemmas raised by genomics and anticipatory governance. The authors noted that it would be useful to carry out future research into how the perception of different scenarios is affected by participant background and training ^78^.

Scenario-based engagement methods can be used on a large scale ^78^, however, in the context of genomics, they have been typically used to engage small groups (less than 100 people) ^56, 78^. Their effectiveness is difficult to assess as to date these studies have collected qualitative data via focus groups or workshops after the scenarios have been presented, limiting the feasibility of collecting data from a large sample.

As we reviewed the literature on purported novel methods, we observed that dialogic and deliberative methods can help public audiences think through and contribute to debates on legal, ethical, and social issues in genomics, and governance considerations.

Two additional examples of innovative public dialogue projects about genomics that were not directly picked up in our literature search, but we feel still provide interesting insights include: A ScienceWise project in England to engage the public in a dialogue about genomic medicine consisting of several in-person events with nearly 100 members of the public and 30 experts. At the final event for this project, a “Genomic Summit”, participants rotated through stations and interacted with different items such as fake newspaper headlines, real-world consent forms for genomic studies, and other props to help them engage in the topic and discuss their perspective with others ^79^. Similarly, a deliberative citizen forum in Belgium gathered 32 members of the public for three weekends to discuss ethical, legal and social issues related to genomics. Activities included role-playing exercises, journalism, making mood boards, debates, and prioritisation exercises where participants voted using tokens ^80^.

These projects both demonstrate the potential for interaction to occur not just amongst participants or between participants and researchers, but also with physical objects and settings. However, both projects required self-motivation and time from the public participants, which may be difficult for those who are disinterested or disengaged. Although different methods have been used to engage the public with genetics and genomics, there is limited evidence as to their effectiveness and impact ^18, 19^. Furthermore, there is no standard measure of effectiveness ^27, 28^ - for example, some engagement methods may be concerned with increasing participation in research or impact on policy, while others may simply aim to increase the opportunity for meaningful connection between scientists and publics. While reach was often reported in the studies that used novel engagement methods, other measures of the quality or impact of engagement were typically not collected. This reflects similar findings from the broader public engagement literature ^27^. The evidence that is available suggests there may be a trade-off between the degree to which methods are interactive and encourage deep engagement, versus the ability to reach many participants when devising public engagement activities. 

## Discussion

This narrative review aimed to set the scene regarding public attitudes toward and engagement with genetics and genomics. We sought to explore novel approaches that have moved public engagement in this area beyond deficit models to a focus on meaningful, bi-directional communication between all stakeholders. We hoped to identify examples of applied public engagement strategies designed specifically for disengaged audiences. However, this field is still rapidly evolving; at this stage, we cannot catalogue definitive, evidence-based approaches for engagement, but rather highlight areas for further development. From the literature we sourced there is insufficient evidence to draw any conclusions about the novelty of the engagement method and its relationship with the ability to convert a disengaged person into an engaged one. Due to lack of evidence, we are also unable to confirm if the novel engagement methods highlighted in this review do indeed provide a method for reaching disengaged audiences – it is distinctly possible that the public audiences who did connect for the engagement activity were already feeling excited and ‘connected to’ science. What is clear from our review is that the behavioural construct of ‘engagement’ was elusive in the published work and certainly no validated measure of this construct was visible. This provides fertile ground for future work.

### What constitutes public engagement on complex topics like genetics and genomics?

Although the theoretical underpinnings of public engagement have moved beyond the deficit model of science communication, in practice public education is still synonymous with public engagement for many. However, this perspective is not limited to genetics and genomics; it has been identified as a wider problem across the sciences ^81, 82^. This has stimulated more detailed explorations of the types of public engagement activities that can take place, including the motivations and expectations of those who deliver and engage with them ^82^. However, if we take the responsible research and innovation approach, then our focus narrows to engagement activities that aim to facilitate meaningful conversation and mutual respect and understanding between all stakeholders ^21^. Indeed, if the purpose of public engagement is to present science that is worthy of a conversation or build trusting relationships so that there is a sense of agency in being able to contribute to policy discussions, public audiences do not necessarily need to be educated about the technicalities of the science. What they do need is clear messaging around *why* they should care and what the specific relevance is to their lives, so that they can make choices about *whether* to engage.

This is a difficult ask when the context has no clear “call to action” and nothing specific is being asked of people (i.e., the engagement is around the conversation as opposed to a motive to increase participation in research, increase uptake of testing or answer a specific policy question). While public attitudes towards genetics and genomics in the UK are mixed, they are also nuanced and dependent on specific contexts and frameworks that govern research and clinical care. Compounding this, those who currently feel disengaged from genetics and genomics are not a homogeneous group and it thus may not be possible to offer a single public engagement approach that cuts across whole populations.

Studies from the UK and internationally consistently point to the importance of addressing inequalities in healthcare and science more broadly to encourage choice about participation in genetics and genomics research. This is particularly needed to address historical and ongoing injustices and inequalities that continue to impact participation today. Without addressing these underlying issues, there is a risk that the application of genomic technology in research and genomic medicine will perpetuate existing inequalities in healthcare and participation in research ^6^. There is limited information available on how to best engage audiences that are currently not connected to the science, whether in research or genomic medicine. And no explicit guidance on whether it is important to tailor make specific engagement strategies to specific demographic groups. However, addressing historical injustices, building, and maintaining trustworthy science and healthcare, and setting up safeguards around consent, privacy and data management appear to be key. Drawing together the attitude literature, we have identified that using public engagement with the aim of reaching some level of public consensus, is likely to be misjudged. When collating outcomes, across different public engagement experiences, it may be more important to draw out shared public values instead of seeking agreement of attitudes. Particularly for contentious subjects like genome editing of human embryos, it may never be possible to agree a national public position, let alone a global one, but this does not mean public engagement is pointless. There are many important outcomes of public engagement that collectively provide a temperature gauge on whether society and science are in sync or at odds.

Also important is determining how public engagement can be sustained beyond an initial activity given the identified importance of creating a feedback loop for participants of all backgrounds, but particularly those from marginalised groups, to recognise their contribution and demonstrate how it has influenced research, clinical processes, or policy-making ^82, 83^ There is an ethical imperative to engage with the public about genetics and genomics, and this engagement should be conceptualised not as a single point of contact, but an extended reciprocal relationship that facilitates the development of trust ^84, 85^. This is underpinned by the need for transparent sharing of knowledge, extending the recognised obligation of researchers to disseminate their research findings to the sharing of mutual learning obtained through public engagement ^86– 89^.

The landscape of public engagement methods used around genomics has no doubt improved over the years as more novel engagement techniques have emerged ^18, 24, 71^. However, there is no single public engagement strategy that we have uncovered that is uniformly effective in engaging populations that have been excluded from genomics research ^18, 19^. From our review of the literature, we start to see a picture emerging which supports a rationale for very well-defined, tailor-made engagement strategies, delivered to clearly defined public groups. These strategies must consider low genomic literacy as well as the need to explain *why* public audiences might want to embrace an engagement opportunity and what they will gain from this (e.g. a voice in shaping policy? an influence in the way science is done? to have an entertaining experience? to increase knowledge and understanding? to gain confidence in understanding the ethical issues raised by science?) All of these possible outputs need to be clearly defined and measured and it would be unreasonable to expect every engagement event to address all of them. It is also important to anticipate and take account of very reasonable suspicion and mistrust of the technology.

While it is evident that creative forms of public engagement are providing novel and valuable insights into how public audiences engage with genomics ^18, 24, 40, 46, 48, 50, 70, 72, 73, 78^, studies rarely explore the intersection of public engagement strategies and disengaged populations. From the limited evidence on effectiveness that is available, there may be trade-offs between the reach of novel engagement methods, and the depth of engagement that can be achieved. For instance, methods that reach a wide audience (e.g., social media) may not be able to collect the same type of information as detailed engagement through scenario-based methods. However, combining different novel methods of engagement may help achieve the desired reach, while also ensuring that participants engage meaningfully, and that this engagement makes a difference in terms of changing policy, for example. 

As some populations do not readily choose to engage with the science of genomics, there is a lack of evidence around what would encourage disengaged audiences to feel that they could connect, if they so choose. However, the findings described above point to the importance of addressing wider inequalities in healthcare, science, and research as a starting point. Building confidence and engagement practices worthy of public trust, and an appreciation of what trustworthy science and healthcare looks like, is key in addressing these inequalities and supporting participation in both genetic medicine and research ^58, 60^ and healthcare providers have an important role in helping to foster trustworthy practices ^61^. Additionally, addressing commonly held concerns (e.g., around data security, privacy, discrimination), and tailor-making engagement approaches which acknowledge these concerns, may encourage disengaged groups to want to become more connected. Whilst outside of the parameters of this narrative review, we also acknowledge that there is a complex relationship between science and society where genetics (inheritance, family, connectedness) is culturally important and highly valued, and communities/publics engage positively with each other about this (e.g.the Deaf culture), without seeking out engagement with science or scientists. 

Across the breadth of novel public engagement events we’ve highlighted, participants chose (no one was forcing them!) to express themselves through the chosen medium, whether that be writing, poetry, film, theatre, social media. This indicates that the process of self-expression may have served some personal needs (an enjoyment of expression? a therapeutic experience? A chance to be heard? A chance to make a difference to both science and society?) When looking across all the public engagement methods we see public audiences taking up the opportunity to express their views directly. Their contribution is gifted back to us in good faith, and we have a moral duty to bring these voices into the public domain so that they are not just heard by the researchers, but (with consent) by wider society. Given our difficulty with finding published records of excellence in public engagement, we suggest that funders of public engagement activities have a duty to make it mandatory for their efforts to be published in searchable formats so that scholarship in this space is not lost and can be built upon. But more importantly, public audiences who give up their time (often for free) to participate in conversation about genomics, should be afforded the courtesy of being offered a clear route to impact. 

### Limitations of the narrative review

We are fully reliant, when searching for published literature, on the words that authors use to describe their work in titles and abstracts. Unless a peer reviewer specifically picks up and challenges these terms then the author can choose subjective terms that may or may not be perceived the same way by others. For example, we searched in both the peer-reviewed and grey literature for articles on public engagement around genomics that also included any of the following terms: *innovative, novel, creative, non-traditional, interactive*, – all these terms are subjective and open to interpretation. Thus, our search managed to pick up articles that we may (with our own subjective bias) feel are not objectively novel. Working in the field of public engagement around genomics we feel there is more published literature present on engagement from the last five years, but unfortunately, we have not been able to source it using the search terms and snowballing we have used. We are also aware that whilst public engagement activities may be written up and presented on a website, they may not be easy to find if search engine optimisation is low. We are therefore left with an intuitive sense that our narrative review, whilst very thorough, has missed important work because the work itself was coded according to different search criteria, or its search engine optimisation meant it could not easily be located.

## Data Availability

No data are associated with this article.

## References

[1] HM Government: Genome UK: the future of healthcare. 2020. Reference Source

[2] Genomics England: About Us. 2022; [04 August 2022]. Reference Source

[3] NHS England: 100,000 Genomes Project. [04 August 2022]. Reference Source

[4] NHS England: NHS Genomic Medicine Service.[04 August 2022]. Reference Source

[5] Lemke AA Harris-Wai JN : Stakeholder engagement in policy development: challenges and opportunities for human genomics. *Genet Med.* 2015;17(12):949–957. 10.1038/gim.2015.8 25764215 PMC4567945

[6] Fatumo S Chikowore T Choudhury A : A roadmap to increase diversity in genomic studies. *Nat Med.* 2022;28(2):243–250. 10.1038/s41591-021-01672-4 35145307 PMC7614889

[7] Kapadia D Zhang J Salway S : Ethnic Inequalities in Healthcare: A Rapid Evidence Review.NHS Race & Health Observatory,2022. Reference Source

[8] Sciencewise: Tomorrow's tech, today: What the public think about five emerging technologies and opportunities for future engagement. 2022. Reference Source

[9] Department of Health & Social Care: The NHS Constitution for England. [05 January 2023]. Reference Source

[10] Timmermans S Buchbinder M : Patients-in-waiting: Living between sickness and health in the genomics era. *J Health Soc Behav.* 2010;51(4):408–23. 10.1177/0022146510386794 21131618

[11] The National Centre for Social Research: British Social Attitudes: The 36th Report.J. Curtice, Clery, E., Perry, J., Phillips M. and Rahim, N., Editor, London,2019.

[12] European Commission: Special Eurobarometer 516 - European citizens' knowledge and attitudes towards science and technology. UK Fact Sheet,2021. Reference Source

[13] BEIS (Department for Business, Energy and Industrial Strategy): Public attitudes to science 2019: Main report. Research Paper Number 2020/012, 2019. Reference Source

[14] British Science Association: Science and British culture (infographic). 2015. Reference Source

[15] Reincke CM Bredenoord AL van Mil MH : From deficit to dialogue in science communication: The dialogue communication model requires additional roles from scientists. *EMBO Rep.* 2020;21(9): e51278. 10.15252/embr.202051278 32748995 PMC7506985

[16] Seethaler S Evans JH Gere C : Science, Values and Science Communication: Competencies for Pushing Beyond the Deficit Model. *Sci Commun.* 2019;41(3):378–88. 10.1177/1075547019847484

[17] Middleton A Milne R Thorogood A : Attitudes of publics who are unwilling to donate DNA data for research. *Eur J Med Genet.* 2019;62(5):316–323. 10.1016/j.ejmg.2018.11.014 30476628 PMC6582635

[18] Holmes L Cresswell K Williams S : Innovating public engagement and patient involvement through strategic collaboration and practice. *Res Involv Engagem.* 2019;5(1): 30. 10.1186/s40900-019-0160-4 31646001 PMC6802177

[19] Nunn JS Tiller J Fransquet P : Public involvement in global genomics research: A scoping review. *Front Public Health.* 2019;7: 79. 10.3389/fpubh.2019.00079 31024880 PMC6467093

[20] Fraaije A van der Meij MG Kupper F : Art for public engagement on emerging and controversial technologies: A literature review. *Public Underst Sci.* 2022;31(6):694–710. 10.1177/09636625221093213 35570661

[21] Bauer A Bogner A Fuchs D : Rethinking societal engagement under the heading of Responsible Research and Innovation: (Novel) requirements and challenges. *J Responsible Innov.* 2021;8(3):342–363. 10.1080/23299460.2021.1909812

[22] Middleton A Costa A Milne R : The Legacy of Language: What we say, and what people hear, when we talk about genomics. *Human Genetics and Genomics Advances.* 2023. 10.1016/j.xhgg.2023.100231 PMC10589723 37869565

[23] National Co-ordinating Centre for Public Engagement: Genome Editing Public Engagement Synergy (GEPES): Resource Guide.National Co-ordinating Centre for Public Engagement, Wellcome Genome Campus and Wellcome, 2019. Reference Source

[24] Ahmedien DAM : New-media arts-based public engagement projects could reshape the future of the generative biology. *Med Humanit.* 2021;47(3):283–291. 10.1136/medhum-2020-011862 32467304

[25] Sperber NR Carpenter JS Cavallari LH : Challenges and strategies for implementing genomic services in diverse settings: experiences from the Implementing GeNomics In pracTicE (IGNITE) network. *BMC Med Genomics.* 2017;10(1): 35. 10.1186/s12920-017-0273-2 28532511 PMC5441047

[26] Beyond the Formal Mechanisms of Public Engagement. Communicating Biobanking Research with Other Means.| Beltrame | TECNOSCIENZA. * Italian Journal of Science & Technology Studies.* 2022.

[27] Boivin A L’Espérance A Gauvin FP : Patient and public engagement in research and health system decision making: A systematic review of evaluation tools. *Health Expect.* 2018;21(6):1075–1084. 10.1111/hex.12804 30062858 PMC6250878

[28] Chapman R : Genetics: International Public Knowledge, Perceptions and Engagement.Goldsmiths, University of London,2020;297. Reference Source

[29] Greenhalgh T Thorne S Mastered K : Time to challenge the spurious hierarchy of systematic over narrative reviews. *Eur J Clin Invest.* 2018;48(6): e12931. 10.1111/eci.12931 29578574 PMC6001568

[30] Boell SK Cecez-Kecmanovic D : A Hermeneutic Approach for Conducting Literature Reviews and Literature Searches. *Communications of the Association for Information Systems.* 2014;34(12). 10.17705/1CAIS.03412

[31] Dixon-Woods M Cavers D Agarwal S : Conducting a critical interpretive synthesis of the literature on access to healthcare by vulnerable groups. *BMC Med Res Methodol.* 2006;6(1):35. 10.1186/1471-2288-6-35 16872487 PMC1559637

[32] Ouzzani M Hammady H Fedorowicz Z : Rayyan—a web and mobile app for systematic reviews. *Syst Rev.* 2016;5(1):210. 10.1186/s13643-016-0384-4 27919275 PMC5139140

[33] VERBI Software: MAXQDA 2022 [computer software].Berlin, Germany,2021. Reference Source

[34] Calabrò GE Sassano M Tognetto A : Citizens' Attitudes, Knowledge, and Educational Needs in the Field of Omics Sciences: A Systematic Literature Review. *Front Genet.* 2020;11: 570649. 10.3389/fgene.2020.570649 33193671 PMC7644959

[35] Hann KEJ Freeman M Fraser L : Awareness, knowledge, perceptions, and attitudes towards genetic testing for cancer risk among ethnic minority groups: a systematic review. *BMC Public Health.* 2017;17(1):503. 10.1186/s12889-017-4375-8 28545429 PMC5445407

[36] Middleton A Milne R Almarri MA : Global Public Perceptions of Genomic Data Sharing: What Shapes the Willingness to Donate DNA and Health Data? *Am J Hum Genet.* 2020;107(4):743–752. 10.1016/j.ajhg.2020.08.023 32946764 PMC7536612

[37] Samuel GN Farsides B : Genomics England’s implementation of its public engagement strategy: Blurred boundaries between engagement for the United Kingdom’s 100,000 Genomes project and the need for public support. *Public Underst Sci.* 2018;27(3):352–364. 10.1177/0963662517747200 29241419 PMC5841566

[38] Chapman R Likhanov M Selita F : New literacy challenge for the twenty-first century: genetic knowledge is poor even among well educated. *J Community Genet.* 2019;10(1):73–84. 10.1007/s12687-018-0363-7 29589204 PMC6325037

[39] Driver MN Kuo SIC Dick DM : Genetic feedback for psychiatric conditions: Where are we now and where are we going. *Am J Med Genet B Neuropsychiatr Genet.* 2020;183(7):423–432. 10.1002/ajmg.b.32815 32812348 PMC8108123

[40] Haran J O’Riordan K : Public knowledge-making and the media: Genes, genetics, cloning and Mass Observation. *Eur J Cult Stud.* 2018;21(6):687–706. 10.1177/1367549416682971

[41] Holden C Bignell L Mukhopadhyay S : The public perception of the facilitators and barriers to implementing personalized medicine: A systematic review. *Per Med.* 2019;16(5):409–420. 10.2217/pme-2018-0151 31591926

[42] McCaughey T Budden DM Sanfilippo PG : A Need for Better Understanding Is the Major Determinant for Public Perceptions of Human Gene Editing. *Hum Gene Ther.* 2019;30(1):36–43. 10.1089/hum.2018.033 29926763

[43] Boardman FK Sadler C Young PJ : Newborn genetic screening for spinal muscular atrophy in the UK: The views of the general population. *Mol Genet Genomic Med.* 2018;6(1):99–108. 10.1002/mgg3.353 29169204 PMC5823674

[44] Hassan L Dalton A Hammond C : A deliberative study of public attitudes towards sharing genomic data within NHS genomic medicine services in England. *Public Underst Sci.* 2020;29(7):702–717. 10.1177/0963662520942132 32664786 PMC7539600

[45] Ballard LM Horton RH Fenwick A : Genome sequencing in healthcare: understanding the UK general public’s views and implications for clinical practice. * Eur J Hum Genet.* 2020;28(2):155–164. 10.1038/s41431-019-0504-4 31527856 PMC6974627

[46] Faure MC Wonkam A Vries JD : Using the Drama of DNA approach to community engagement in genomic research in South Africa: experiences and lessons learnt [version 1; peer review: 1 approved, 1 approved with reservations]. *AAS Open Res.* 2020;3:1–8. 10.12688/aasopenres.13045.1

[47] Delhove J Osenk I Prichard I : Public Acceptability of Gene Therapy and Gene Editing for Human Use: A Systematic Review. *Hum Gene Ther.* 2020;31(1–2):20–46. 10.1089/hum.2019.197 31802714

[48] Platt T Platt J Thiel D : Engaging a state: Facebook comments on a large population biobank. *J Community Genet.* 2017;8(3):183–197. 10.1007/s12687-017-0302-z 28382416 PMC5496840

[49] Milne R Morley KI Howard H : Trust in genomic data sharing among members of the general public in the UK, USA, Canada and Australia. *Hum Genet.* 2019;138(11–12):1237–1246. 10.1007/s00439-019-02062-0 31531740 PMC6874520

[50] Boll J : The sum of our parts: the voices of the Human Genre Project. *Eur J Engl Stud.* 2018;22(3):317–330. 10.1080/13825577.2018.1513702

[51] Jones KH Daniels H Squires E : Public views on models for accessing genomic and health data for research: Mixed methods study. *J Med Internet Res.* 2019;21(8): e14384. 10.2196/14384 31436163 PMC6727690

[52] Wilkins CH : Precision medicine for everyone. *NEJM Catalyst.* 2018;4(1). Reference Source

[53] Fox K : The Illusion of Inclusion — The “All of Us” Research Program and Indigenous Peoples’ DNA. *N Engl J Med.* 2020;383(5):411–413. 10.1056/NEJMp1915987 32726527

[54] Mapes BM Foster CS Kusnoor SV : Diversity and inclusion for the *All of Us* research program: A scoping review. *PLoS One.* 2020;15(7): e0234962. 10.1371/journal.pone.0234962 32609747 PMC7329113

[55] Silva P Dahlke DV Smith ML : An Idealized Clinicogenomic Registry to Engage Underrepresented Populations Using Innovative Technology. * J Pers Med.* 2022;12(5):713. 10.3390/jpm12050713 35629136 PMC9144063

[56] Miah A : Nanoethics, Science Communication, and a Fourth Model for Public Engagement. *Nanoethics.* 2017;11(2):139–152. 10.1007/s11569-017-0302-9 28845203 PMC5554473

[57] Rosas LG Nasrallah C Park VT : Perspectives on precision health among racial/ethnic minority communities and the physicians that serve them. *Ethn Dis.* 2020;30(Suppl 1):137–148. 10.18865/ed.30.S1.137 32269455 PMC7138446

[58] Kraft SA Doerr M : Engaging populations underrepresented in research through novel approaches to consent. *Am J Med Genet C Semin Med Genet.* 2018;178(1):75–80. 10.1002/ajmg.c.31600 29512940 PMC5910200

[59] Lee SS Cho MK Kraft SA : " *I don't want to be Henrietta Lacks*": diverse patient perspectives on donating biospecimens for precision medicine research. *Genet Med.* 2019;21(1):107–113. 10.1038/s41436-018-0032-6 29887604 PMC6289900

[60] Sabatello M Callier S Garrison NA : Trust, Precision Medicine Research, and Equitable Participation of Underserved Populations. *Am J Bioeth.* 2018;18(4):34–36. 10.1080/15265161.2018.1431328 29621444 PMC5890957

[61] Persaud A Bonham VL : The Role of the Health Care Provider in Building Trust Between Patients and Precision Medicine Research Programs. *Am J Bioeth.* 2018;18(4):26–28. 10.1080/15265161.2018.1431327 29621443 PMC6604055

[62] Sharif SM Blyth M Ahmed M : Enhancing inclusion of diverse populations in genomics: A competence framework. *J Genet Couns.* 2020;29(2):282–292. 10.1002/jgc4.1263 32250032

[63] Mulrine S Blell M Murtagh M : Beyond trust: Amplifying unheard voices on concerns about harm resulting from health data-sharing. *Med Access Point Care.* 2021;5: 23992026211048421. 10.1177/23992026211048421 36204496 PMC9413596

[64] Middleton A Hewison J Mueller RF : Attitudes of deaf adults toward genetic testing for hereditary deafness. *Am J Hum Genet.* 1998;63(4):1175–80. 10.1086/302060 9758618 PMC1377492

[65] Fry A Littlejohns TJ Sudlow C : Comparison of Sociodemographic and Health-Related Characteristics of UK Biobank Participants with Those of the General Population. *Am J Epidemiol.* 2017;186(9):1026–1034. 10.1093/aje/kwx246 28641372 PMC5860371

[66] Hopkins H Kinsella S Evans G : Implications of whole genome sequencing for newborn screening: A public dialogue. Hopkins Van Mil: London,2021. Reference Source

[67] National Co-ordinating Centre for Public Engagement: What is public engagement?2020; [04 August 2022]. Reference Source

[68] Global Citizens’ Assembly on Genome Editing: ABOUT Globalca. [04 August 2022]. Reference Source

[69] Participedia: Australian Citizens’ Jury on Genome Editing. [04 August 2022]. Reference Source

[70] Brieger K Zajac GJM Pandit A : Genes for Good: Engaging the Public in Genetics Research via Social Media. *Am J Hum Genet.* 2019;105(1):65–77. 10.1016/j.ajhg.2019.05.006 31204010 PMC6612519

[71] Farrell M Wilkinson C : A reappraisal of public engagement in Oxford during the pandemic: three case studies. *Res Involv Engagem.* 2022;8(1):10. 10.1186/s40900-022-00343-z 35346388 PMC8960072

[72] Mayeur C Saelaert M Van Hoof W : The Belgian DNA Debate: An Online Deliberative Platform on the Ethical, Legal, and Social Issues of Genomics. *Public Health Genomics.* 2021;24(3–4):149–159. 10.1159/000515356 33951658

[73] Howell AA Reisenauer KN Valkanas MM : Beyond the Base-ics: approaches to driving connection through genetics in museums. *Journal of Science Communication.* 2022;21(1):A02. 10.22323/2.21010202

[74] Reinsborough M : Art-Science collaboration in an EPSRC/BBSRC-funded synthetic biology UK research centre. *Nanoethics.* 2020;14(1):93–111. 10.1007/s11569-020-00367-3 32435319 PMC7228991

[75] Sturgis P Brunton-Smith I Fife-Schaw C : Public attitudes to genomic science: an experiment in information provision. *Public Underst Sci.* 2010;19(2):166–180. 10.1177/0963662508093371 20533796

[76] Gorman R Farsides B : Writing the worlds of genomic medicine: experiences of using participatory-writing to understand life with rare conditions. *Med Humanit.* 2022;48(2):e4. 10.1136/medhum-2021-012346 35418508 PMC9185826

[77] Mass Observation: Genes, Genetics & Cloning. 2006. Reference Source

[78] Lehoux P Miller FA Williams-Jones B : Anticipatory governance and moral imagination: Methodological insights from a scenario-based public deliberation study. *Technol Forecast Soc Changea.* 2020;151:119800. 10.1016/j.techfore.2019.119800

[79] Ipsos MORI: A public dialogue on genomic medicine: time for a new social contract. 2019; [04 August 2022]. Reference Source

[80] Sciensano: My DNA Everybody’s Business? Qualitative analysis of the Belgian Citizen Forum on the Use of Genomic Information. 2020; [04 August 2022]. Reference Source

[81] Calice MN Bao L Beets B : A triangulated approach for understanding scientists’ perceptions of public engagement with science. *Public Underst Sci.* 2023;32(3):389–406. 10.1177/09636625221122285 36154528

[82] Scheufele DA Krause NM Freiling I : What we know about effective public engagement on CRISPR and beyond. *Proc Natl Acad Sci U S A.* 2021;118(22): e2004835117. 10.1073/pnas.2004835117 34050014 PMC8179128

[83] Farooqi A Jutlla K Raghavan R : Developing a toolkit for increasing the participation of black, Asian and minority ethnic communities in health and social care research. *BMC Med Res Methodol.* 2022;22(1):17. 10.1186/s12874-021-01489-2 35026996 PMC8758375

[84] Solomon MZ Gusmano MK Maschke KJ : The Ethical Imperative And Moral Challenges Of Engaging Patients And The Public With Evidence. *Health Aff (Millwood).* 2016;35(4):583–9. 10.1377/hlthaff.2015.1392 27044955

[85] Russell J Fudge N Greenhalgh T : The impact of public involvement in health research: what are we measuring? Why are we measuring it? Should we stop measuring it? *Res Involv Engagem.* 2020;6(1):63. 10.1186/s40900-020-00239-w 33133636 PMC7592364

[86] Cremin H Aryoubi H Hajir B : Post-abyssal ethics in education research in settings of conflict and crisis: Stories from the field. *Br Educ Res J.* 2021;47(4):1102–1119. 10.1002/berj.3712

[87] Robinson-Pant A Singal N : Researching ethically across cultures: issues of knowledge, power and voice. *Compare: A Journal of Comparative and International Education.* 2013;43(4):417–421. 10.1080/03057925.2013.797719

[88] Smith R : Publishing research from developing countries. *Stat Med.* 2002;21(19):2869–77. 10.1002/sim.1291 12325103

[89] Chandler R Anstey E Ross H : Listening to Voices and Visualizing Data in Qualitative Research: Hypermodal Dissemination Possibilities. *SAGE Open.* 2015;5(2): 2158244015592166. 10.1177/2158244015592166

