# Supplementary material for: Public engagement with genomics
Source: Wellcome Open Res. 2023 Sep 18;8:310. Originally published 2023 Jul 18. [Version 2] doi: 10.12688/wellcomeopenres.19473.2 (PMC10624956; doi:10.12688/wellcomeopenres.19473.2)
Supplement: Peer review history

# Reviewer report: Reviewer response for version 2

**DOI:** 10.21956/wellcomeopenres.22194.r68233

**Published:** 2023-11-03

**Authors:** Selina Goodman, Leigh Jackson

**Affiliations:** 1 College of Medicine & Health, University of Exeter, Exeter, England, UK

This clear and comprehensive review addresses how public engagement contributes to the quality of research and can mitigate against inequalities of care between different sectors of the population. We would agree that a “failure to engage” does significantly reduce the potential benefits. The authors recognise that people have a right to choose and a right to decline participation but as they correctly state, the level of disengagement with this type of discourse is likely to perpetuate and deepen social inequalities and further potentially weaken the validity of research findings in genomics and genetics. Perhaps the title could be a little more encompassing of the content explored. Even a simple tweak such as “Public disengagement with genomics” may be a better hook for readers as the content of this excellent manuscript is more focussed on how to engage those who are not actively connected.

 The introduction is comprehensive and sets the scene well for the review to come. Why this issue is important, how it has been considered before and existing issues in the field are well described.

 The short paragraph on pressure to publish and other existing strategies, whilst very important and true seemed a little out of place as it stands; maybe a little expansion would help? Pressure from whom? Funders, health services, researchers? Probably all of these but it would help to spell it out a bit. Perhaps mentioning that public engagement is too often viewed by many (funders and researchers alike) as a bit of a box ticking exercise and that only by mandating reporting of these activities in publications or other outputs will these activities be given the importance they deserve. As the authors state, the current model often leads to public engagement being at the end of the process and simply giving information, which is sub-optimal. We note that this is covered towards the end of the discussion so maybe less needs adding here however a little expansion would still be helpful.

 Also “we are unaware…” rather undersells the expertise of the authors; a casual reader may take this to mean they have not looked adequately when in fact the truth is that these issues have been considered and sought by members of this group for many years and if such strategies existed they would certainly be aware of them. This could be made a little more explicit?

 The authors use a good metaphor for the engagements of publics, likening this to walking away or choosing to look away from opportunities to engage with science. While they talk about people actively seeking out or building “active connections” with science, it is unclear what those active connections to science might look like? The authors posit that possibly the majority of the UK population are disengaged with genomics and science in general, which may be true but they could have recognised that some well publicised events spark increased engagement, such as when the actress Angelina Jolie wrote about her experiences of genetic testing in 2013. In addition, the reasons that may contribute to negative views or suspicion around genetics are important to define because that brings insight into how this distrust might be addressed. However, the authors do emphasise that their focus in this review is on dialogue with diverse publics in this context which would seek to provide understanding of what specifically fuels disengagement around genetics within given social groups.

 The aims of the narrative are clear and focused.

 The methodology of the review is clear and valid but one small suggestion: the fact that a systematic review of studies was not carried out was clear, but perhaps a short sentence on why that wasn’t deemed appropriate for what the authors describe as a niche field? To be clear, we think the current approach as absolutely appropriate but that readers may benefit from understanding a little more of the decision-making process here.

 Another small point possibly worthy of discussion here given the discussion section on lack of representation. The search was restricted to English language studies. Whilst appropriate for the UK context being investigated and also due to practical considerations when searching in other languages, even with translation services, perhaps this should be mentioned as a limitation. The authors correctly discuss later that White Europeans are the predominant enrolled participants in genomic studies but in only focussing on English language studies, those underserved publics who may have been studied by non-English-speaking researchers will again be excluded, as will the lessons from those researchers. Language is a significant barrier to engagement and novel strategies to reach these communities are likely to involve dialogs in their preferred language.

 The attitudes towards genomics in clinic and research contexts were well described and possible reasons for these attitudes posited. Reasons why audiences may be disengaged were clearly discussed and informative. Use of specific examples of lack of trust due to historical abuses or recent perception were particularly helpful to give context to these reasons.

 It was striking that a recurrent theme within many of studies was that researchers did not make explicit what they intended to do with their findings. We believe that this observation could feed into or reinforce the scepticism around the value of research participation reported by some marginalised groups, would the authors like to comment on this?

 The arts-based engagement section was very helpful in clearly delineating some of the issues with public engagement activities where they don’t deliver a two-way dialog, despite potentially having the ability to facilitate such an exchange. This really highlights the importance of evaluation and planning activities based on what the researchers want to achieve early-on. Knowing which data to collect is extremely useful in helping to consider activity outcomes and how they may be achieved.

 In the section on the potential for discrimination, the review highlighted an important finding that certain groups had experienced discrimination within the NHS which indicated to them that the NHS was not fit to handle sensitive information. It is also sobering to learn that underrepresented people’s involvement in GWAS studies has been “stagnating or decreasing”, which gives even greater impetus to the need for wider public engagement.

 The discussion is an accurate representation of the findings, caveated where appropriate due to lack of evidence and makes clear suggestions for future work and progress.

 Possibly a personal preference, but ending on the limitations feels a little anti-climactic and the exclusion of papers not written in English needs comment with the this section due to the  potential exclusion of some relevant literature relating to ethnic minorities where research participation in English would be a significant barrier. Perhaps a short conclusion paragraph to pull everything together may be appropriate so that there is a final paragraph which read in a more positive way about how these findings could be taken forward? However, this paper does provide an overview of why the disengaged publics, due to their disengagement, rarely have a voice and may continue to struggle to influence research, policy or how science is applied unless more innovative public engagement strategies are used.

 Overall, a very clearly written, well conducted review which gives a timely overview of the public engagement space and provides though-provoking suggestions for future research and policy direction.

Is the review written in accessible language?

Yes

Are all factual statements correct and adequately supported by citations?

Yes

Are the conclusions drawn appropriate in the context of the current research literature?

Yes

Is the topic of the review discussed comprehensively in the context of the current literature?

Yes

Reviewer Expertise:

Dr Leigh Jackson conducts research into ethics of genomics research, public health genomics, cancer susceptibility genomics, adult and newborn  genomic screening. Dr Selina Goodman conducts research into family communication around genetic diagnoses.

We confirm that we have read this submission and believe that we have an appropriate level of expertise to confirm that it is of an acceptable scientific standard.

# Reviewer report: Reviewer response for version 2

**DOI:** 10.21956/wellcomeopenres.22194.r67153

**Published:** 2023-10-05

**Authors:** Mavis Machirori

**Affiliations:** 1 Ada Lovelace Institute, London, UK

Thank you for the authors for engaging extensively with earlier comments. This paper was much smoother and easier to read, comments were clearer and the arguments made suggest the continual need for clarity in what is novel, who is disengaged and how to measure the impact of any public engagement activities within genetics/genomics.

Is the review written in accessible language?

Yes

Are all factual statements correct and adequately supported by citations?

Yes

Are the conclusions drawn appropriate in the context of the current research literature?

Yes

Is the topic of the review discussed comprehensively in the context of the current literature?

Partly

Reviewer Expertise:

NA

I confirm that I have read this submission and believe that I have an appropriate level of expertise to confirm that it is of an acceptable scientific standard.

# Reviewer report: Reviewer response for version 1

**DOI:** 10.21956/wellcomeopenres.21570.r63755

**Published:** 2023-08-31

**Authors:** Mavis Machirori

**Affiliations:** 1 Ada Lovelace Institute, London, UK

- This article is about public engagement activities within genomics. It is an enjoyable paper that is
  - Based on the need to attend to concerns of under-representation or exclusion or disengagement in genomic research
  - Takes a narrative approach and
    - Provides a description and summary of evidence or discourse from literature
    - Makes observations about gaps and suggests new strategies for engaging with audiences
  - Refreshing to see arguments that argue for going beyond science communication particularly in complex areas like genomics
  - So, this article is not just about pe in general as one might first understand it, but with a specific focus on those who are disengaged, further reading suggests this is about what is known about the disengaged, their attitudes first and then what methods have been used to improve dialogue/engagement with them (It was a little difficult to get the flow of the aims in the introduction and the methods as the way they are written seemed contradictory at times.)
  - I would suggest authors just need to specifically say if the article is about disengaged groups rather than publics in general. Which would make point 1 in methods not necessary to articulate - I sometimes found it  difficult to know if what is referred is disengaged or publics in general.
- In their introduction,
  - authors highlight some themes that make pe important and why they will be important in the context of GenomeUK
    - e.g. Clinically important in reducing inequalities, benefits being shared with those excluded *but I think there is another fundamental reason for engagement - to see if the research question bodes well with the public and this means pe work should be part of the design of the studies rather than after a piece of work has begun - should be stressed more in the strategies and should be addressed*
    - *Also what if the ones excluded have no interest in that particular area - p.e. Needs to set out how to hold that tension and not overpromise if policy directions differ from immediate community sector concerns - would be great if this was addressed too*
  - Not so clear who is referred to as we in the 'why we might car about this' - scientists? Publics?
  - The section on '1:17 people' did not seem to sit fluently here - not sure it adds much in this section
- Methods
  - The methods section could do with a little more summary detail on the following
    - *Why is international evidence not included if it linked to a specific country?*
    - *A brief explanation of their theoretical approach would be good - what makes an interpretive narrative review that draws on hermeneutic reviews, hermeneutics circles and interpretive synthesis - a good approach. What is it? And how does it move from the data to the themes discussed?*
- Attitudes research - I appreciate that the authors engage with the complex reasons people make decisions about research, that is not always rooted in perceived benefits but that there are data-based decisions which incorporate events related to data use
  - In clinical care
    - Should be an acknowledgement that while cost may not seem an issue in access to gene therapies, the NHS funding model via NICE shows that therapies might still be restricted, and authors should account for this to complete a critique of their findings
    - While no country specific research was included in the criteria, if making comparisons, would also be useful to know if this was similar across all health models internationally or there were some areas where difference could be attributed to other factors
  - While this might not be possible with the results they already collected - I wonder, was there also any difference noted in the general public research about a high level difference in attitudes by different groups - they do this for the audiences disengaged but are there times when those same groups are highly engaged (or is this a gap in the literature that we actually don't know when those groups are highly engaged, either because we (researchers/academics etc) don't have the data, have not asked them or have disaggregated publics only when it comes to negative attitudes)). Such insight would show when people are interested but the structure of the system (pathways etc do not cater to them, rather than the reason being inherently to do with the people). It also helps us recognise when it is structural issues rather than people's attitudes that lead to disengagement.
- Disengagement
  - There is literature that suggests nuance about religion and ethnicity (separately, with Arabic communities in Australia, and with African American communities in the US, e.g. Which suggests interests in genomics - which the authors missed. This literature could add depth to the issues of disengagement beyond whether it is Western science or not). While this is out of scope of the parameters they set, it would be good to see an acknowledgement of this in their discussion). This helps to move the narrative beyond disengagement along demographics of religion or ethnicity as well.
- Novel approaches
  - It is interesting that the review does not generally seem to tackle demographic groups, because of a lack of info, which would have helped us understand the gap highlighted by one of the aims on describing the scale of disengagement. How do the authors' findings now respond to what we want to know about how to engage the disengaged, because one could argue that those who participated in the novel approaches were already interested and not the audience of concern for this narrative work?
    - E.g if by religious groups or by ethnicity, is there something we could say to attract those groups e.g. Do ethnic groups use social media more and would that be novel for them?

 An otherwise enjoyable read with important observations about how engagement can happen for it to be meaningful. I think minor revisions will strengthen some of their arguments and critique.

Is the review written in accessible language?

Yes

Are all factual statements correct and adequately supported by citations?

Yes

Are the conclusions drawn appropriate in the context of the current research literature?

Yes

Is the topic of the review discussed comprehensively in the context of the current literature?

Partly

Reviewer Expertise:

NA

I confirm that I have read this submission and believe that I have an appropriate level of expertise to confirm that it is of an acceptable scientific standard, however I have significant reservations, as outlined above.

# Response

**Published:** 2023-09-08

**Authors:** Anna Middleton

**Affiliations:** Wellcome Trust Sanger Institute, UK

Thank you so much for this very constructive and helpful review; your comments have helped us to adapt and improve the article for clarity and so we are hugely grateful for this. I’ll address each of your points below:

*This article is about public engagement activities within genomics. It is an enjoyable paper that is*

-
  - *Based on the need to attend to concerns of under-representation or exclusion or disengagement in genomic research*
  - *Takes a narrative approach and*
  - *Provides a description and summary of evidence or discourse from literature*
  - *Makes observations about gaps and suggests new strategies for engaging with audiences*
  - *Refreshing to see arguments that argue for going beyond science communication particularly in complex areas like genomics*

Thank you for this feedback.

-
  - So, this article is not just about pe in general as one might first understand it, but with a specific focus on those who are disengaged, further reading suggests this is about what is known about the disengaged, their attitudes first and then what methods have been used to improve dialogue/engagement with them (It was a little difficult to get the flow of the aims in the introduction and the methods as the way they are written seemed contradictory at times.)

I've added in additional text to help with the flow of the aims into the introduction.  

-
  - *I would suggest authors just need to specifically say if the article is about disengaged groups rather than publics in general. Which would make point 1 in methods not necessary to articulate - I sometimes found it  difficult to know if what is referred is disengaged or publics in general.*

It’s very helpful to have this feedback as it indicates that we have not been clear enough in our definitions of ‘broad public audiences’ and the relationship between this and ‘disengaged audiences’. We actually make the argument that these are likely to be the same group. The government report from the Department for Business, Energy, and Industrial Strategy on Public Attitudes to Science from 2019 and also the British Science Association indicate that the majority of the public say they are not actively connected to science, nor do they talk to anyone about science – in our view, this defines them as ‘disengaged’. Both of these reports indicate that while the public says they ‘value’ science and they are positive about its existence, the data suggests that irrespective of this, they still feel a personal disconnection to it. The reports indicate that publics see science as something that ‘scientists do’ as opposed to something that publics feel they can be part of. It is this lack of perceived relevance and connection that is an indicator to us that public engagement opportunities need to do so much more help people see the connections to their lives. I’ve added in multiple pieces of additional text throughout the paper to hopefully make this message clearer.  

- *In their introduction,*
  - *authors highlight some themes that make pe important and why they will be important in the context of GenomeUK*
    - *e.g. Clinically important in reducing inequalities, benefits being shared with those excluded but I think there is another fundamental reason for engagement - to see if the research question bodes well with the public and this means pe work should be part of the design of the studies rather than after a piece of work has begun - should be stressed more in the strategies and should be addressed* 

I’ve added in text that reflects this in the Introduction.

-
  -
    - *Also what if the ones excluded have no interest in that particular area - p.e. Needs to set out how to hold that tension and not overpromise if policy directions differ from immediate community sector concerns - would be great if this was addressed too*

We have already addressed this in our previous paper that has just been published and is now referenced in the text: Middleton et al (2023) ‘The Legacy of Language: What we say, and what people hear, when we talk about genomics’ Human Genetics and Genomics Advances https://doi.org/10.1016/j.xhgg.2023.100231

-
  - Not so clear who is referred to as we in the 'why we might car about this' - scientists? Publics?

I’ve changed this to: “However, these rights can only be exercised regarding genomic medicine if there is some level of awareness of why we (as publics, patients and future patients) might care about this”.

  

-
  - The section on '1:17 people' did not seem to sit fluently here - not sure it adds much in this section

Apologies, I can’t locate exactly what this is referring to, but hopefully the new additions to the text clarify the point you were highlighting.

  

- Methods
  - The methods section could do with a little more summary detail on the following
    - *Why is international evidence not included if it linked to a specific country?*

I’ve added in this additional text: "While our focus is on the UK, international evidence is included where it does not focus on a specific country. Given the public engagement landscape is very broad internationally, with no universally accepted definition of public engagement itself, we wanted this specific narrative review to start with the literature most meaningful to the UK context. However, given that novel engagement methods are infrequently labelled as ‘novel’ in the literature, we included those that were indexed with this term, regardless of country. See Table 1 for inclusion and exclusion criteria."  

-
  -
    - *A brief explanation of their theoretical approach would be good - what makes an interpretive narrative review that draws on hermeneutic reviews, hermeneutics circles and interpretive synthesis - a good approach. What is it? And how does it move from the data to the themes discussed?*

I’ve added this text in: “We chose this approach to give us the broadest overview possible of a niche field that uses different methodological approaches in both research and engagement delivery.” The theorectical approach moves the data to the themes discussed and is explained in this existing text: “We did not seek to provide a comprehensive and systematic review of studies in these areas, but rather to describe [the data], in broad terms [the themes], the types of peer-reviewed research and engagement that have been undertaken to make recommendations for further areas of development.”

  

- *Attitudes research - I appreciate that the authors engage with the complex reasons people make decisions about research, that is not always rooted in perceived benefits but that there are data-based decisions which incorporate events related to data use*
  - *In clinical care*
    - *Should be an acknowledgement that while cost may not seem an issue in access to gene therapies, the NHS funding model via NICE shows that therapies might still be restricted, and authors should account for this to complete a critique of their findings*

I couldn’t quite see how to make this fit in the paper. We don’t go into the details of what specific NHS services are on offer, nor mention any NHS funding models via NICE as we are deliberately keeping the subject of genetics as broad as possible, to include both research and clinical applications.

-
  -
    - *While no country specific research was included in the criteria, if making comparisons, would also be useful to know if this was similar across all health models internationally or there were some areas where difference could be attributed to other factors*

Agree this would be useful to know. But we didn’t include country specific research in the selection criteria as we wanted to hone in on the UK. Across country comparisons could be the focus of future work.

-
  - *While this might not be possible with the results they already collected - I wonder, was there also any difference noted in the general public research about a high level difference in attitudes by different groups - they do this for the audiences disengaged but are there times when those same groups are highly engaged (or is this a gap in the literature that we actually don't know when those groups are highly engaged, either because we (researchers/academics etc) don't have the data, have not asked them or have disaggregated publics only when it comes to negative attitudes)). Such insight would show when people are interested but the structure of the system (pathways etc do not cater to them, rather than the reason being inherently to do with the people). It also helps us recognise when it is structural issues rather than people's attitudes that lead to disengagement.*

Agree this would be fascinating to explore. Unfortunately, we are unable to do this with the data we gathered.

  

- *Disengagement*
  - *There is literature that suggests nuance about religion and ethnicity (separately, with Arabic communities in Australia, and with African American communities in the US, e.g. Which suggests interests in genomics - which the authors missed. This literature could add depth to the issues of disengagement beyond whether it is Western science or not). While this is out of scope of the parameters they set, it would be good to see an acknowledgement of this in their discussion). This helps to move the narrative beyond disengagement along demographics of religion or ethnicity as well.*

I’ve expanded the section on ‘Scale of Disengagement’ and also expanded the Discussion to add a little more depth to the critique of disengagement.

  

- *Novel approaches*
  - *It is interesting that the review does not generally seem to tackle demographic groups, because of a lack of info, which would have helped us understand the gap highlighted by one of the aims on describing the scale of disengagement. How do the authors' findings now respond to what we want to know about how to engage the disengaged, because one could argue that those who participated in the novel approaches were already interested and not the audience of concern for this narrative work?*
    - *E.g if by religious groups or by ethnicity, is there something we could say to attract those groups e.g. Do ethnic groups use social media more and would that be novel for them?*

There are serious limits to the way published literature describes audience demographics, which means it is not possible to draw out exactly who is disengaged and why. This is why we’ve included the attitude literature as this does provide demographic data. From the review we’ve done we are not able to draw any conclusions about whether different engagement strategies would work for specific demographic groups (as the data just isn’t there to guide us). But to make this a bit clearer in the text I’ve added in the following to the Discussion: “There is limited information available on how to best engage audiences that are currently not connected to the science, whether in research or genomic medicine. And no explicit guidance on how to tailor make specific engagement strategies to specific demographic groups.” *An otherwise enjoyable read with important observations about how engagement can happen for it to be meaningful. I think minor revisions will strengthen some of their arguments and critique.*   Thank you!
